# Activation of *ABCC* Genes by Cisplatin Depends on the CoREST Occurrence at Their Promoters in A549 and MDA-MB-231 Cell Lines

**DOI:** 10.3390/cancers14040894

**Published:** 2022-02-11

**Authors:** Maciej Sobczak, Magdalena Strachowska, Karolina Gronkowska, Agnieszka Robaszkiewicz

**Affiliations:** 1 Department of General Biophysics, Faculty of Biology and Environmental Protection, University of Lodz, Pomorska 141/143, 90-236 Lodz, Poland; maciej.sobczak@edu.uni.lodz.pl (M.S.); magdalena.strachowska@edu.uni.lodz.pl (M.S.); karolina.gronkowska@edu.uni.lodz.pl (K.G.); 2 Bio-Med-Chem Doctoral School of the University of Lodz and Lodz Institutes of the Polish Academy of Sciences, University of Lodz, Banacha 12/16, 90-237 Lodz, Poland

**Keywords:** CoREST, ABC-family transporters, multidrug-resistance, EP300, p53

## Abstract

**Simple Summary:**

Cisplatin resistance is a common issue that affects patients with a variety of cancers who are treated with this drug. In this research, we present a novel epigenetic mechanism that controls the expression of ABC-family transporters, which are involved in multidrug resistance. We report that the CoREST complex may be a key factor that determines the transcription of ABC transporters in non-small cell lung and triple-negative breast cancer cells (A549 and MDA-MB-231, respectively) treated with cisplatin. By occupying gene promoters, this multi-subunit repressor prevents both an EP300-dependent increase in ABCC transcription induced by the alkylating drug and gene overexpression in cisplatin-resistant phenotypes. Moreover, the CoREST-free promoter of ABCC10 responds to cisplatin with EP300-mediated gene activation, which is only possible in p53-proficient cells.

**Abstract:**

Although cisplatin-based therapies are common among anticancer approaches, they are often associated with the development of cancer drug resistance. This phenomenon is, among others, caused by the overexpression of ATP-binding cassette, membrane-anchored transporters (ABC proteins), which utilize ATP to remove, e.g., chemotherapeutics from intracellular compartments. To test the possible molecular basis of increased expression of ABCC subfamily members in a cisplatin therapy mimicking model, we generated two cisplatin-resistant cell lines derived from non-small cell lung cancer cells (A549) and triple-negative breast cancer cells (MDA-MB-231). Analysis of data for A549 cells deposited in UCSC Genome Browser provided evidence on the negative interdependence between the occurrence of the CoREST complex at the gene promoters and the overexpression of *ABCC* genes in cisplatin-resistant lung cancer cells. Pharmacological inhibition of CoREST enzymatic subunits—LSD1 and HDACs—restored gene responsiveness to cisplatin. Overexpression of CoREST-free *ABCC10* in cisplatin-resistant phenotypes was caused by the activity of EP300 that was enriched at the *ABCC10* promoter in drug-treated cells. Cisplatin-induced and EP300-dependent transcriptional activation of *ABCC10* was only possible in the presence of p53. In summary, the CoREST complex prevents the overexpression of some multidrug resistance proteins from the ABCC subfamily in cancer cells exposed to cisplatin. p53-mediated activation of some *ABCC* genes by EP300 occurs once their promoters are devoid of the CoREST complex.

## 1. Introduction

Cisplatin was approved as an anti-cancer drug in 1987; however, cisplatin-based therapies are still commonly used against a variety of malignancies such as lymphoma, lung, bladder, ovarian, cervical as well as head and neck cancers [1]. For non-small cell lung cancer (NSCLC), cisplatin-based treatment is often the only effective approach, as 85–90% of NSCLC patients lack mutations that allow for specific attack cancer cells in targeted therapy [2]. Similarly, the lack of a molecule-specific cure for triple-negative breast cancer (TNBC) patients imposes the need to apply conventional chemotherapy. Although cisplatin is not considered a first-line treatment against TNBC, growing attention has been paid to cisplatin combinations with other agents. This drug prevents DNA replication and gene transcription by forming cross-links between alkylated DNA bases [3]. In response to DNA adducts, ATM (ataxia telangiectasia mutated) and ATR (ATM and Rad3-related) kinases undergo activation, phosphorylate CHK1/CHK2 and p53, thereby triggering death and growth inhibition of cancer cells [4]. Although this alkylating agent emerged to be effective against solid tumors, its use is often associated with the occurrence of severe side effects, including nausea, vomiting, and nephro- and ototoxicity. Moreover, the phenomenon of cisplatin resistance is a serious issue. While some cancers may be naturally resistant to this drug, others might decline their responsiveness over the course of anti-cancer treatment [5]. Loss of cell vulnerability to drugs is quite complex and involves changes in cancer cell biology at many levels. The mechanism of cisplatin resistance is frequently associated with alterations in drug transport through the cellular membrane, drug detoxication, enhanced or lowered effectiveness of DNA damage repair, inhibition of apoptosis, and overexpression of anti-apoptotic proteins [6]. In particular, efflux of cisplatin was reported to be mediated by increased glutathione concentration (GSH), overexpressed glutathione-S-transferase (GST), and ABC-family (ATP-binding cassette) transporters in cisplatin-resistant NSCLC. GST catalyzes conjugation of cytotoxic drug and GSH, and then the less toxic complex is transported outside the cancer cell by ABC family members [7].

ABC transporters utilize energy from ATP hydrolysis to shuttle a variety of small biological compounds such as ions, nutrients, and drugs through the cellular membrane [8]. Some of these proteins are overexpressed in a variety of drug-resistant cancers. These include ABCB1 (glycoprotein P, MDR1), ABCC1 (MRP1—multidrug resistance protein 1), ABCC2 (MRP2), ABCC4 (MRP4), ABCC5 (MRP5), and ABCG2 (BCRP—breast cancer resistance protein) in cisplatin-resistant cells [9,10,11,12,13,14,15]. Only three of these—ABCC2, ABCC5, and ABCC6—were reported to actively transport cisplatin out of the cell [16]. This suggests that cisplatin-induced overexpression of ABC genes may provide cancer cell resistance to other drugs. Moreover, ABC transporters reveal low substrate specificity.

Transcription efficiency of *ABC* genes is elevated in response to stressors such as oxidative stress or drug-related toxicity, transcription factors, e.g., NF-kB, Nrf2, p53, chromatin remodeling enzymes as well as tyrosine kinase effectors (Akt or ERKs), which transduce signals to chromatin [17,18]. Increased activity of p53, caused by gain-of-function mutations, was documented to enhance the activity of the *ABCB1* promoter in a variety of cancer cell types [19]. In response to UV radiation or different compounds, including chemotherapeutics, the MDR1 enhancesome, which comprises two histone acetylases—P/CAF and EP300 (E1A binding protein P300)—as well as transcription factors Sp1, Sp3, NF-Y—was found at the promoter of the *ABCB1* gene. Recruitment of the above-mentioned enhancesome to chromatin results in increased chromatin accessibility to transcriptional apparatus due to the histone acetylation by the above-mentioned acetylases [20]. Enzymatic activity of EP300 controls cellular processes such as cell proliferation and DNA repair; hence, it plays an important role in tumorigenesis [21,22]. Some reports indicate that EP300 acetylates, stabilizes, and activates p53 overexpression of which inhibits caspase 9 in the human lung cancer cell line H1299, thereby allowing these cells to avoid cisplatin-induced apoptosis [23,24]. More recent papers also indicate another functional link between these two proteins, where p53 overexpression in H1299 promotes self-acetylation and, hence, increased activity of EP300 and elevated acetylation of H3K18 [25]. Moreover, identification of chromatin regions enriched in EP300 indicated the recruitment of acetylase to p53-driven promoters in a p53-dependent manner in human lung cancer cells [26]. These findings suggest the possible implication of p53-EP300 mutual interdependence in the development of multi-drug resistance. Particularly, EP300 bromodomain inhibitor, I-CBP112, recently emerged as an *ABC* gene repressor in p53 wild-type cell lines [27].

In contrast to EP300, histone deacetylases (HDACs) that oppose acetylase activity may inhibit some ABC genes, as treatment with HDAC inhibitors increased *ABCB1* expression [28]. Similar to HDACs, LSD1 (lysine-specific demethylase—also known as KDM1A) is capable of suppressing ABC-family gene expression upon deficiency of EP300 activity. Inhibition of LSD1 counteracts suppression of *ABCC1* and *ABCC10* expression caused by EP300 bromodomain inhibitor [27].

Although LSD1 removes transcription-promoting chromatin signatures by erasing methyl groups from di- and monomethylated lysine 4 of histone 3, it requires the CoREST (REST corepressor 1 or RCOR) to function on a nucleosome and not only histones [29]. The latter protein links LSD1 with HDAC1/2 activity since the two histone deacetylases associated with REST (RE1-silencing transcription factor) and Sin3 form a core of a chromatin remodeling complex, which is responsible for the repression of numerous genes [30,31]. Considering the above-mentioned examples of HDAC and LSD1 contribution to downregulation of some ABC gene promoter activity, the involvement of the CoREST complex in the regulation of multi-drug resistance seems possible. Moreover, the referred papers highlight the role of EP300 and p53 in controlling the cellular level of protein transporters functionally associated with an active drug efflux, thus their potential involvement in the development of cisplatin resistance in cancer cells.

These premises prompted us to test the possible molecular link between CoREST complex occurrence at the promoters of the ABCC gene subfamily of ABC transporters and their overexpression in cisplatin-resistant cancer cells. For this purpose, we generated two cisplatin-resistant cell lines derived from non-small cell lung cancer cells (A549) and triple-negative breast cancer cells (MDA-MB-231), where cisplatin is considered in mono- or combined neo- or adjuvant chemotherapy. Furthermore, we compared the interaction of EP300 and p53 with CoREST-bound and -free *ABCC* gene promoters with their responsiveness to cisplatin.

## 2. Materials and Methods

### 2.1. Materials

The A549 cell line was purchased from ATCC. The MDA-MB-231 cell line was purchased from Sigma-Aldrich. DMEM High Glucose w/L-Glutamine w/Sodium Pyruvate, fetal bovine serum, and antibiotics (penicillin and streptomycin) were purchased from Biowest (CytoGen, Zgierz, Poland). Lipofectamine RNAiMAX, OptiMem, Dynabeads™ Protein G, High-Capacity cDNA Reverse Transcription Kit, PageRuler™ Prestained Protein Ladder (10 to 180 kDa), SuperSignal™ West Pico Chemiluminescent Substrate, TRI Reagent™, Pierce™ Protease Inhibitor Tablets (EDTA-free; PIC), Goat anti-Mouse IgG (H+L) Secondary Antibody, HRP (#32430), SlowFade™ Glass Soft-set Antifade Mountant (with DAPI), anti-MRP10 (ABCC10) Polyclonal Antibody (#PA5101678), UltraPure™ Phenol:Chloroform:Isoamyl Alcohol (25:24:1, *v*/*v*) (#15593031), TaqMan™ universal master mix II), TaqMan™ gene expression assays: ABCC3 (Hs00978452_m1), ABCC4 (Hs00988721_m1), ABCC10 (Hs01056200_m1), ACTB (Hs01064292_g1), GAPDH (Hs02786624_g1), and TBP (Hs99999910_m1) were purchased from Thermo Fisher Scientific (Thermo Fisher Scientific, Warsaw, Poland). ChIP-grade antibodies: anti-ABCC3 (#D8V8J), anti-ABCC4 (#D2Q20), anti-LSD1 (#2139) normal rabbit IgG (#2729), p53 (#7F5) anti-histone H3 (#1B1B2), anti-H3K27ac (#D5E4), and anti-EP300 (#D2X6N) were purchased from Cell Signaling Technology (LabJOT, Warsaw, Poland). iEP300 (C646, #10549) and cisplatin (#13119) were purchased from Cayman Chemical (Biokom, Janki/Warsaw, Poland). Nunc™ Lab-Tek™ Chamber Slide was ordered from Biokom, Janki/Warsaw, Poland. L15 Medium, oligonucleotides for real-time PCR, Sodium butyrate (303410-5G), KAPA SYBR^®^ FAST Universal 2×, Anti-CoREST Antibody (#07-455), resazurin sodium salt were ordered from Sigma-Aldrich (Poznan, Poland). For transient gene silencing, siRNA Control (sc-37007) and siRNA P53 (sc-29435) were purchased from Santa Cruz Biotechnology (AMX, Lodz, Poland).

### 2.2. Cell Culture and Treatment with Inhibitors

A549 cells were cultured in DMEM supplemented with 10% FBS, penicillin–streptomycin (50 U/mL and 50 µg/mL, respectively) in 5% CO_2_. MDA-MB-231 cells were initially cultured in L15 medium supplemented with 15% FBS, penicillin–streptomycin (50 U/mL and 50 µg/mL, respectively) without CO_2_ equilibration. After five passages, cells were adapted to grow in DMEM supplemented with 10% FBS, penicillin–streptomycin (50 U/mL and 50 µg/mL, respectively) in 5% CO_2_. For both cell lines, culture medium was replaced twice a week.

Before treatment cells were seeded on culture plates and maintained at the logarithmic growth phase, the compounds: C646 (5 µM; iEP300), cisplatin (10 µM), and sodium butyrate (0.1 mM, iHDAC) were added to cells in a single dose for 24 h prior to analysis. All the concentrations, as well as incubation times, were assessed based on our own results.

### 2.3. Cisplatin Resistance Induction

A549 and MDA-MB-231 cells were cultured in DMEM supplemented with 10% FBS, penicillin–streptomycin (50 U/mL and 50 µg/mL, respectively) in 5% CO_2_. Both cell lines were seeded into 25 cm^3^ culture bottles. In the period of 5 months, depending on the cell proliferation status (approximately every 3 weeks), the cells were treated with 10 µM cisplatin, since it is within the cisplatin therapeutic range of 1000–5000 µg/L [32]. Cells were incubated with the compound for 48 h. After incubation, the medium containing cisplatin was removed, and cells were washed with PBS and replaced with fresh cisplatin-free medium twice a week.

### 2.4. Resazurin Toxicity Assay

Non-resistant and cisplatin-resistant cells were seeded in 96-well plates and treated with varying concentrations of cisplatin (0; 0.01; 0.1; 0.5; 1; 5; 10; 25; 50; 100 (μM)), doxorubicin (0; 0.01; 0.025; 0.05; 0.1; 0.2; 0.3; 0.4; 0.5; 1 (μM)) and paclitaxel 0; 0.0005; 0.001; 0.005; 0.01; 0.1; 0.5; 1; 5; 10 (μM)). After 48 h incubation with the drugs, resazurin solution (5 μM) was added to the plates and incubated for 4 h in 37 °C. Next, fluorescence indicating the number of living cells was measured with a fluorescence microplate reader (BioTek Synergy HTX, Biokom, Poland) at 530ex/590em nm. The fluorescence value of untreated cells was assumed as 100%.

### 2.5. Quantification of Gene Expression

For the sake of gene expression evaluation, mRNA was extracted from cells using TRI Reagent™. Afterward, mRNA was reverse transcribed with the High-Capacity cDNA Reverse Transcription Kit. cDNA fragments were amplified in real-time PCR (KAPA SYBR^®^ FAST Universal 2× or TaqMan™ universal master mix II); CFX96 C1000 Touch, BioRad Warsaw, Poland) using the following primer pairs: ABCC10 Forward: 5′ CGGGTTAAGCTTGTGACAGAGC 3′, Reverse: 5′ AACACCTTGGTGGCAGTGAGCT 3′; ABCC2 Forward: 5′ AATCAGAGTCAAAGCCAAGATGCC 3′, Reverse: 5′ TAGCTTCAGTAGGAATGATTTCAGGAGCAC 3′; ABCC3 Forward: TCCTTTGCCAACCTTTCTCTGCAACTAT, Reverse: 5′ CTGGATCATGTCTGTCAGATCCGT 3′; ABCC4 Forward: 5′ TGATGAGCCGTATGTTTTGC 3′, Reverse: 5′ CTTCGGAACGGACTTGACAT 3′; ABCC5 Forward: 5′ AGAGGTGACCTTTGAGAACGCA 3′, Reverse: 5′ CTCCAGATAACTCCACCAGACGG 3′; ABCG2 Forward: 5′ CCGCGACAGCTTCCAATGACCT 3′, Reverse: 5′ GCCGAAGAGCTGCTGAGAACTGTA 3′. The following housekeeping genes were used for normalization: GAPDH Forward: 5′ TTCTTTTGCGTCGCCAGCCGA 3′, Reverse 5′ GTGACCAGGCGCCCAATACGA 3′; HPRT1 Forward: 5′ TGACACTGGCAAAACAATGCA 3′. Reverse: 5′ GGTCCTTTTCACCAGCAAGCT 3′; TBP Forward: 5′CACGAACCACGGCACTGATT 3′, Reverse: 5′ TTTTCTTGCTGCCAGTCTGGAC 3′ or TaqMan™ gene expression assays. mRNA level of particular genes was first normalized to three housekeeping genes. Next, the fold-change of values of each of the housekeeping genes over untreated control was calculated. Finally, the mean of all three-fold change values was calculated and used for statistical analysis.

For evaluating protein expression, cells were lysed in cell lysis buffer (15 mM NaCl/50 mM Tris-HCl/0.5% sodium deoxycholate/0.5% SDS) and sonicated (Bandelin Sonopuls HD2070). Afterward, cell lysates were separated with SDS-PAGE, transferred to nitrocellulose membranes, and stained overnight with primary antibodies (1:5000) at 4 °C. After staining, using HRP-conjugated secondary antibodies (1:5000; room temperature; 1 h), the signal was developed using SuperSignal™ West Pico Chemiluminescent Substrate and acquired with ChemiDoc-IT2 (UVP, Meranco, Poznan, Poland).

### 2.6. Transient Gene Silencing

The day prior to transfection, cells were seeded at a density of 100,000 cells per well. The next day, siRNA-RNAiMAX complexes (prepared in a ratio of 20 nmol siRNA and 3 µL of transfection reagent), suspended in OptiMem medium, were added. After 4 h of incubation with the complexes, DMEM supplemented with 10% FBS, penicillin–streptomycin (50 U/mL and 50 µg/mL, respectively) was added to a total volume of 1 mL.

### 2.7. Chromatin Immunoprecipitation

Cells were fixed by adding formaldehyde to the culture medium. Cells were lysed, and then chromatin was sheared using a sonicator (Bandelin Sonopuls HD2070). Antibodies and Dynabeads™ Protein G, which were pre-blocked with 0.5% BSA/PBS, were added to the obtained supernatant. Samples were incubated overnight on a rotator at 4 °C. The next day, chromatin–antibody–magnetic bead complexes were washed, and chromatin was decrosslinked in 2% SDS/TE buffer at 65 °C overnight. DNA was extracted using phenol:chloroform:isoamyl alcohol (25:24:1). Fragments spanning sites of interest at the selected gene promoters were amplified using KAPA SYBR^®^ FAST Universal 2× and 0.1% DMSO. The following primer pairs surrounding potential p53 binding sites were used: ABCC2 Forward: 5′ AGGTCAAGGCTGCAATGAAT3′, Reverse: 5′CTGTCATCGACCCAACCTTT 3′; ABCC10 Forward: 5′ CTTGTCCAAGGTCATGCAGC 3′, Reverse: 5′GCCCCACGGACAAATAATGC 3′; ABCC3 Forward: 5′ ACTCAATGACTCATCGGCCC 3′, Reverse: 5′ GGCTAACAGTCCAGGAGTCG 3′; ABCC4 Forward 5′ GACCTCAAGCAGGGATGTG 3′, Reverse: 5′ GGCTCCCTTTGGCGTCG 3′.

### 2.8. Confocal Microscopy

For confocal imaging, cells were seeded on a Nunc™ Lab-Tek™ Chamber Slide. In order to stain chromatin-bound fraction of EP300, cells were washed with 1% BSA/PBS and then pre-permeabilized for 5 min in 1% BSA/PBS/0.1% Triton X-100. Afterward, cells were fixed in 4% formaldehyde in PBS for 15 min. After blocking in 5% FBS/1x PBS/0.5% Triton X-100 for 1 h, cells were incubated overnight with primary anti-EP300 antibody (1:400) 1% BSA/PBS/0.5% Triton X-100. After washing with 1% BSA in PBS, cells were incubated with secondary antibody (1:400) 1% BSA/PBS/0,5% Triton X-100 for 2 h in the dark. After washing with PBS, slides were mounted with SlowFade™ Glass Soft-set Antifade Mountant (with DAPI). TCS SP8 (Leica Microsystems, Wetzlar, Germany) with objective 63×/1.40 (HC PL APO CS2, Leica Microsystems, Germany) was used for slide imaging. The following wavelength values of excitation and emission were used for specimen visualization: 485 and 500–550 nm for Alexa Fluor^®^ 488 and 405 and 430–480 nm for DAPI. The average fluorescence was calculated using at least 100 single cells for each sample. Fluorescence intensity was determined as the arbitrary units (a.u.) with Leica Application Suite X (LAS X, Leica Microsystems, Germany).

### 2.9. Transcription Factor Binding Sites Assessment

For the analysis of chromatin interacting proteins, specifically at the promoters (TSS -2kbp) of ABCC2, ABCC3, ABCC5, ABCC10, ABCG2 in A549 cells, track ENCODE 3 TFBS in the UCSC table browser was used. The search was conducted for every transcription factor listed in the aforementioned track for A549 cells. Queries were then submitted to usegalaxy.org.

### 2.10. Statistical Analysis

Data are presented as the mean ± SEM (standard error of the mean), Student’s *t* test or the Mann–Whitney test was used to calculate statistically significant differences between two samples (marked with * when *p*  <  0.05), while one-way analysis of variance (ANOVA) or the Kruskal–Wallis test followed by corresponding post hoc test was carried out to compare multiple samples (marked with * when *p* < 0.05). Statistics were calculated using GraphPad Prism 8.01 software. The decision on using the parametric versus non-parametric test was conducted after testing Gaussian distributions of data with the Shapiro–Wilk test.

## 3. Results

### 3.1. Non-Small Cell Lung Cancer Cells Exposed to Multiple Doses of Cisplatin Gain Resistance to Drugs of Various Chemical Structures and Activity

**Figure 1:**
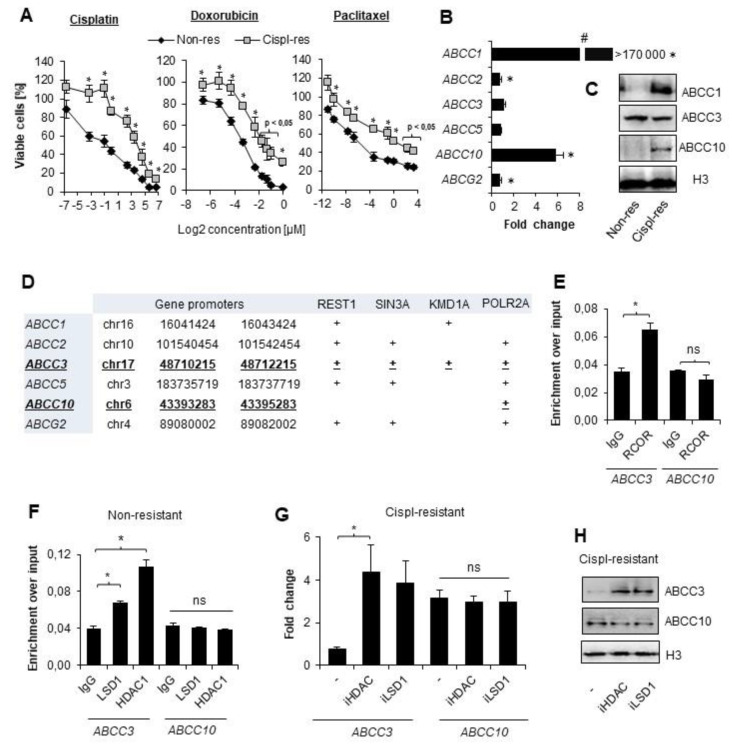
CoREST complex represses genes that are not overexpressed in cisplatin-resistant lung cancer cells. (**A**) Vulnerability to wide concentration range of cisplatin, doxorubicin, and paclitaxel was compared between non-resistant (non-res) and cisplatin-resistant (cispl-res) A549 cells. Viability was evaluated with resazurin-based assay. Metabolic activity of untreated cells was assumed as 100%. (**B**) Fold-change in mRNA level of some *ABCC* and *ABCG2* genes was measured by real-time PCR using TaqMan assays. Transcription of transporter genes was normalized first to housekeeping genes (ACTB, GAPDH, and TBP), and then, mRNA levels of *ABCC* and *ABCG2* were assumed as 1. (**C**) Protein level of ABCC1, ABCC3 and ABCC10 was compared in lysates of non-resistant and cispl-resistant A549 cells by Western blot. Histone H3 was used as a loading control. Original WB can be found at Figure S1. (**D**) The indicated promoter regions (TSS ± 2 kbp) were searched for transcription factors, chromatin remodeling enzymes, and POLR2A in Genome Browser database (assembly: GRCh37/hg19, group: Regulation, track: ENCODE 3 TFBS, table: A549 ATF3—ZBTB33). (**E**,**D**) Occurrence of REST (**E**) as well as LSD1 and HDAC1 (**F**) at the promoters of ABCC3 and ABCC10 was tested by ChIP-qPCR. Amplified promoter regions spanned p53 binding site. Ten percent input was used as an internal control. (**G**) Functional impact of LSD1 and HDAC1 occurrence at the *ABCC3* gene promoter was evaluated by treating cispl-resistant A549 cells with LSD1 and pan-HDAC inhibitors (0.1 µM SP2509 and 250 µM sodium butyrate, respectively) for 24 h and measuring gene mRNA level by real-time PCR. Results were normalized to housekeeping genes, and then, mRNA level of non-resistant untreated cells was assumed as 1. (**H**) Effect of HDAC and LSD1 inhibition on ABCC3 and ABCC10 protein level was monitored by Western blot in cisplatin-resistant A549 cells. Histone H3 was used as a loading control. Protein bands were measured by densitometry using ImageJ, and numerical data are available in Table S2. (**A**,**B**,**E**–**G**) All of the bars represent the mean of replicates ± SEM. (**A**,**B**) Difference between survivability of cisplatin-resistant and non-resistant cells and in expression of particular ABC transporters were analyzed with the *t* test or Mann–Whitney test, depending on their Gaussian distribution. (**E**) Differences in RCOR enrichment at the promoters of interest were analyzed with the *t* test. (**F**) The effect of iHDAC and iLSD1 on *ABCC3* and *ABCC10* expression in non-resistant A549 was analyzed with ANOVA and the Dunnett’s post hoc test. (**G**) The impact of HDAC and LSD1 inhibition on cisplatin-resistant A549 cells was analyzed with ANOVA and Tukey’s test. (**A**,**B**,**E**–**G**) Statistically significant differences between analyzed groups are marked with * when *p* < 0.05. Detailed statistical analysis can be found in Table S1.

In order to assess whether A549 cells developed multidrug resistance, the resazurin assay was conducted. We evaluated varying concentrations of cisplatin, doxorubicin, and paclitaxel to assess if cisplatin-treated cells tolerate higher concentrations of chemotherapeutics than their wild-type counterparts. Cisplatin-resistant A549 cells exhibited better survival rates than the original cell line in the entire concentration range of three tested compounds (Figure 1A). This indicates that multiple treatments of A549 cells with cisplatin allow these cells to gain resistance to chemotherapeutics, which vary in chemical structure and biological activity. Furthermore, to test which ABC-family transporters undergo overexpression in response to cancer cell treatment with an alkylating agent, we compared their mRNA level in cisplatin-resistant versus basal cell lines. As shown in Figure 1B, loss of cell vulnerability to anticancer drugs was associated with strong overexpression of ABCC1 and ABCC10 and a slight but statistically significant increase in ABCC2 and ABCG2 transcription, whereas mRNA levels of ABCC3 and ABCC5 remained unchanged. Importantly, an immense fold increase in ABCC1 may result from a low basal mRNA level of this gene in A549 cells. Visualization of three proteins—ABCC1, ABCC3 and ABCC10—by Western blot confirmed the results obtained by real-time PCR (Figure 1C) and protein abundance of ABCC10 was considerably higher in cisplatin-resistant cells, whereas ABCC3 was comparable between two cell phenotypes.

### 3.2. CoREST Complex Subunits Occupy Promoter of ABCC3 That Remains Transcriptionally Irresponsive to Cisplatin in A549

In search of the rationale behind some of the transporters being overexpressed in cisplatin-resistant A549 cells, we investigated regions of TSS ± 2 kbp for the presence of transcription-regulating factors that may interact with regulatory elements of ABC genes and affect their expression. For this purpose, we used ChIP-seq data of transcription factors deposited in the Genome Browser in an ENCODE 3 TFBS track. As shown in Figure 1D, promoters of ABCC1, ABCC2, and ABCC5 were enriched in individual components of the CoREST complex; however, REST, Sin3, and two enzymatic subunits—HDAC1 and LSD1—were detected only at the promoter of ABCC3 that remained unchanged upon gaining resistance to cisplatin. In contrast, the promoter of ABCC10, which underwent considerable activation by repeated administration of an alkylating drug, was only enriched in POLR2A. This observation led us to hypothesize that the Co-REST repressive complex may interfere with the intensification of some ABCC gene transcription in response to cisplatin. Using ChIP-qPCR, we confirmed that REST complex members—Co-REST as well as LSD1 and HDAC1—are enriched at the ABCC3 promoter region, but not at the ABCC10 promoter (Figure 1E,F). Furthermore, to assess the functional impact of CoREST members on ABC gene expression in cisplatin-resistant A549 cells, we treated them with iLSD1 (0.1 µM SP2509) and pan-HDAC inhibitor (250 µM sodium butyrate) for 24 h and measured mRNA levels of ABCC3 and ABCC10 (Figure 1G). Inhibition of CoREST enzymatically active units did not affect the expression of ABCC10. This supports data in Figure 1E and 1F and confirms the lack of CoREST’s direct impact on transcription activation of ABCC10 by cisplatin. As expected, transcription of ABCC3 was substantially enhanced by both inhibitors in cisplatin-resistant A549 cells. This led to the conclusion that CoREST prevents ABCC3 induction by cisplatin.

### 3.3. EP300 Drives ABCC10 Overexpression in Cisplatin-Resistant A549 Cells

**Figure 2:**
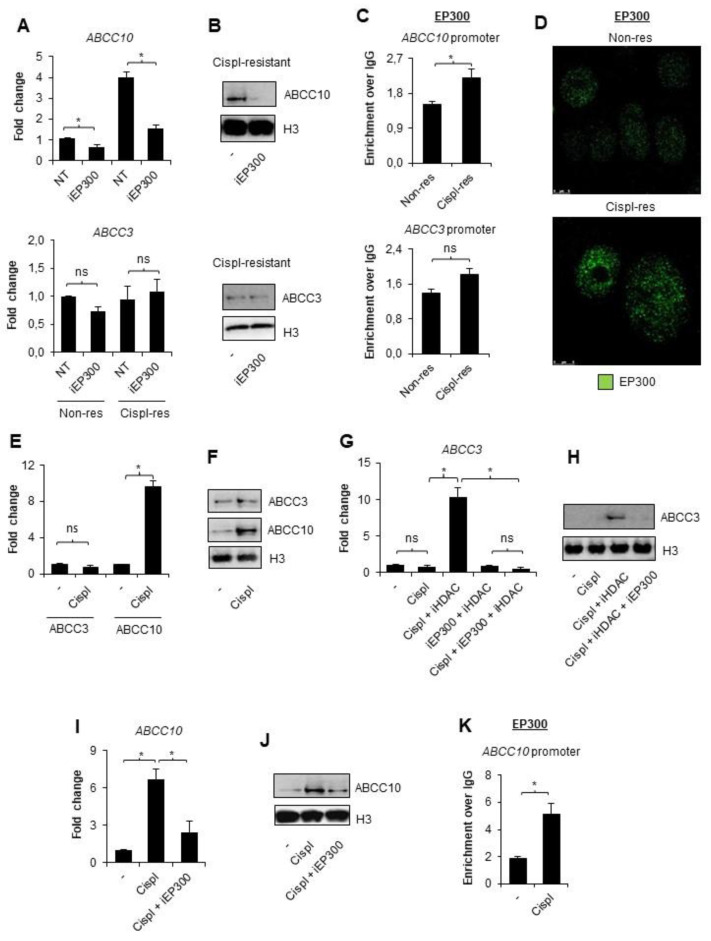
EP300 is responsible for *ABCC10* overexpression in cisplatin-resistant A549 cells. (**A,B**) Contribution of EP300 in transcription of *ABCC3* and *ABCC10* was evaluated by treating non- and cispl-resistant A549 cells with EP300 inhibitor—C646 (5 µM, 24 h)—and quantifying gene mRNA level by real-time PCR (**A**) and protein by Western blot (**B**). (**C**) Recruitment of EP300 to the promoter of *ABCC10* in two A549 phenotypes was monitored by ChIP-qPCR. (**D**) Chromatin-bound EP300 was visualized in non- and cispl-resistant cells by immunocytostaining, followed by confocal microscopy. Green fluorescence derived from Alexafluor488-conjugated secondary antibody corresponds to EP300 appearance in cells. (**E,F**) *ABCC3* and *ABCC10* response to a single dose of cisplatin was measured by non-resistant A549 cell treatment with 10 µM drug for 24 h. (**E**) The treatment was followed by mRNA quantification with TaqMan-based real-time PCR. After initial mRNA normalization to housekeeping genes, expression of *ABCC3* and *ABCC10* was assumed as 1 in untreated cells. (**F**) Changes in ABCC3 protein levels were measured by Western blot. (**G,H**) HDAC-repressive role on the ABCC3 promoter during non-resistant cell single induction with cisplatin was tested by adding pan-HDAC inhibitor (250 µM sodium butyrate) for 1 h prior to anticancer drug. The same approach was utilized to confirm the contribution of EP300 to the observed release of *ABCC3* from silencing upon combination of iHDAC with cisplatin. iEP300—5 µM C646—was added for 1 h prior to cisplatin (together with iHDAC). (**G**) mRNA was extracted from cells 24 h after cell stimulation with the drug, and gene transcription was quantified by TaqMan-based real-time PCR. After normalization to mRNA level of *ACTB*, *GAPDH*, and *TBP*, transcription of *ABCC3* was compared to untreated cells, where the gene transcription was set as 1. (**H**) Impact of HDAC inhibition with subsequent cisplatin administration on ABCC3 protein expression was visualized by Western blot. (**I**) Similarly, the impact of EP300 on *ABCC10* transcription activated by single dose of cisplatin was analyzed by adding non-resistant cells with iEP300 for 1 h prior to chemotherapeutic. The following steps were the same as in (**E**). (**J**) Inhibition of *ABCC10* activation by iEP300 was also visualized at the protein level by Western blot. (**K**) Cisplatin-induced interaction of EP300 with *ABCC10* promoter was monitored by ChIP-qPCR in non-resistant A549 cells exposed to 10 µM cisplatin for 24 h. (**A**,**C**,**E**,**G**,**I**,**K**) All bars represent the mean of replicates ± SEM. (**A**) The effect of iEP300 on expression of *ABCC3* and *ABCC10* in non-resistant and cisplatin-resistant A549 were analyzed with *t* test. (**C**) The change in enrichment of EP300 at promoters of *ABCC3* and *ABCC10* between non-resistant and cisplatin-resistant A549 was analyzed with the Mann–Whitney test. (**E**) The effect of cisplatin on *ABCC3* and *ABCC10* expression was analyzed with *t* test and Mann–Whitney test, respectively. (**G**) The influence of cisplatin, iHDAC, and iEP300 on expression of ABCC3 gene was analyzed with the Kruskal–Wallis test and Dunn’s multiple comparison test. (**I**) The effect of cisplatin and C646 on *ABCC10* expression was evaluated with ANOVA and Tukey’s post hoc test. (**K**) The enrichment of EP300 at ABCC10 gene promoter after exposure to cisplatin was analyzed with the Mann–Whitney test. (**A**,**C**,**E**,**G**,**I**,**K**) Statistically significant differences between analyzed groups were marked with * when *p* < 0.05. Detailed statistical analysis can be found in Table S1. Densitometry results of Western blot images are included in Table S2. Original WB can be found at Figure S1.

Knowing that EP300 forms transcription modulating complexes with HDAC1 at the E2F-driven promoters, which include the ABCC gene subfamily, and bearing in mind previous reports, which indicated the role of PCAF and EP300 in transcription activation of ABCB1, we took into consideration a potential involvement of the latter acetyltransferase in the regulation of ABCC10 gene expression in cisplatin-resistant A549 cells [20,22,33]. To test this hypothesis, we treated both A549 phenotypes with EP300 inhibitor C646 (5 µM) for 24 h (Figure 2A,B). The deficiency of EP300 activity led to a substantial decrease in ABCC10 expression in cisplatin-resistant A549 cells; however, a statistically significant decline in ABCC10 mRNA was also found in the wild type. As expected, ABCC3 was unaffected by EP300 inhibition, as the CoREST complex seemingly blocked its promoter region (Figure 2A,B). Analysis of the two gene promoters by ChIP-qPCR confirmed weak but statistically significant enrichment of EP300 at the ABCC10 promoter, but not ABCC3, in cisplatin-resistant cells (Figure 2C). Of note, ABCC10 responded to EP300 inhibition also in basal A549 cells, thereby suggesting that acetyltransferase may be abundant at the gene promoter also in non-resistant cells. To further validate EP300 occurrence on the chromatin of cisplatin-resistant A549, the chromatin-bound fraction of the enzyme was stained and further imaged under a confocal microscope (Figure 2D). EP300 interaction with chromatin was enhanced in cisplatin-resistant A549 cells.

Since we found that EP300 is more abundant on chromatin in NSCLC cells after long-term exposure to cisplatin, and, particularly, at the ABCC10 promoter that allows for relatively strong overexpression of this gene in cisplatin-resistant phenotype, we decided to test the direct ABCC10 response to a single dose of cisplatin (10 µM). As shown in Figure 2E,F, the expression of ABCC10 was significantly elevated at the mRNA and protein level in cisplatin-treated cells, whereas the expression of ABCC3 remained unchanged. To test whether histone deacetylases, which contribute to CoREST-mediated ABCC3 repression in cisplatin-resistant cells, prevent ABCC3 activation by a single dose of cisplatin, we added a pan-HDAC inhibitor—sodium butyrate—for 1 h prior to cisplatin (Figure 2G,H). Deficiency of HDAC activity led to the cisplatin-induced elevation of ABCC3 transcription. Similar profiles of change were observed for two other ABCC genes: ABCC2 and ABCC5, where HDAC inhibition was necessary to observe cisplatin-induced increase in the gene transcription (Figure S2). Inhibition of EP300 countered the effect of iHDAC and maintained a low level of mRNA. Similarly, EP300 emerged responsible for increased ABCC10 transcription in response to a single dose of cisplatin, since C646 considerably reduced the drug-induced activation of this gene. Accordingly, EP300 was recruited to the ABCC10 promoter in response to the treatment of non-resistant cells with the alkylating drug (Figure 2K).

### 3.4. P53 Allows for EP300-Mediated Increase in ABCC10 Transcription after Single Dose of Cisplatin in Non-Resistant A549 Cells

**Figure 3:**
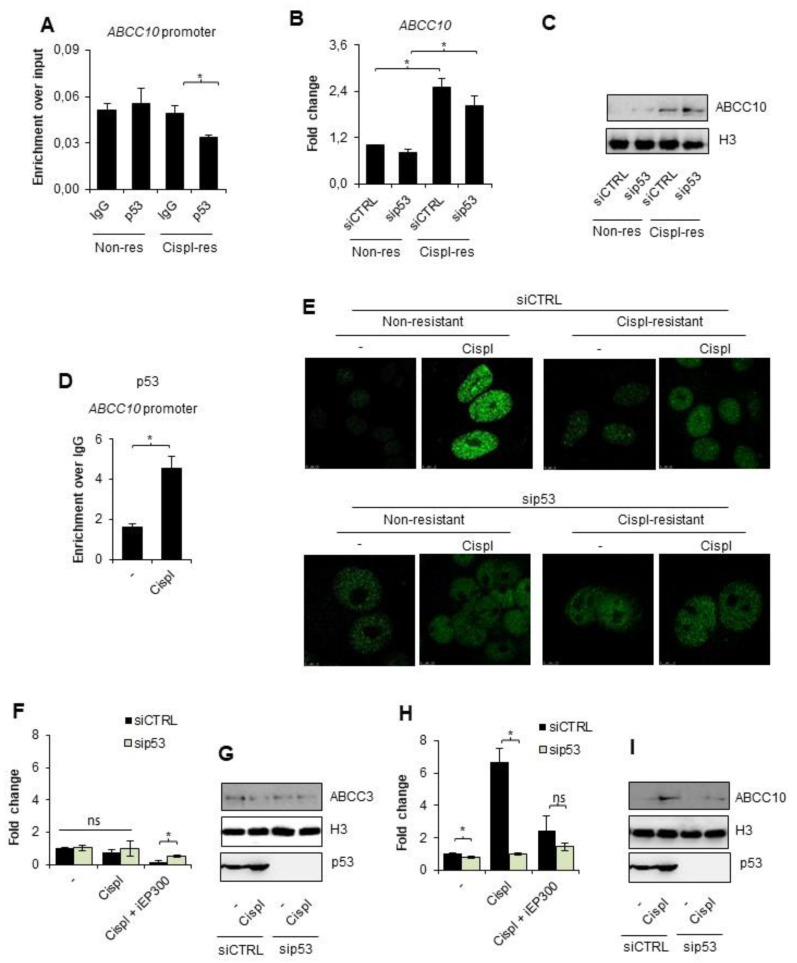
p53 recruits EP300 to chromatin upon exposure of non-resistant A549 cell to cisplatin and controls ABCC10 direct response to the alkylating agent. (**A**) Enrichment of p53 at the promoter of *ABCC10* in unstimulated non- and cisplatin-resistant lung cancer cells was analyzed with ChIP-qPCR. (**B**,**C**) Transient silencing of p53 was utilized to assess the possible impact of this protein on *ABCC10* expression in two phenotypes of unstimulated A549 cells. (**B**) mRNA was quantified 48 h after cell transfection with siRNA by TaqMan-based real-time PCR, normalized to *ACTB*, *GAPDH*, and *TBP* and is shown as fold-change versus siCTRL transfected cells. (**C**) Protein level was compared between siCTRL and sip53 by Western blot 72 h after cell transfection. Histone H3 was used as an internal control. (**D**) Recruitment of p53 to the promoter of *ABCC10* in cells treated with cisplatin (10 µM) for 24 h was analyzed by ChIP-qPCR. (**E**) The role of p53 in EP300 enrichment at chromatin was visualized by immunocytostaining followed by confocal microscopy. Cells were exposed to cisplatin (10 µM) 48 h after cell transfection with siRNA, and EP300 was stained with rabbit antibody 24 h after cell treatment with anticancer drugs. Green fluorescence of Alexaflor488-conjugated secondary antibody marks chromatin-bound acetyltransferase. (**F**–I) Impact of p53 on expression of *ABCC3* (**F**,**G**) and *ABCC10* (**H**,**I**) was evaluated by comparing gene expression in cisplatin-induced non-resistant A549 cells transfected with siCTRL and sip53. mRNA (**F**,**H**) was isolated and quantified by TaqMan-based real-time PCR and protein (**G**,**I**) by Western blot 24 h after administration of the chemotherapeutic (10 µM). After normalization to housekeeping controls, mRNA level in siCTRL untreated cells was assumed as 1. In Western blot, histone H3 was used as loading control. (**A**,**B**,**D**,**F**,**H**) All bars represent the mean of replicates ± SEM. (**A**,**F**,**H**) The enrichment of p53 at the ABCC10 promoter and the effect of cisplatin and iEP300 on *ABCC3* and *ABCC10* gene expression in cells that differ in p53 level were analyzed with *t* test. (**B**) The effect of sip53 silencing on ABCC10 expression in non-resistant and cisplatin resistant A549 was evaluated with Kruskal–Wallis test and Dunn’s multiple comparison test. (**D**) The enrichment of p53 at *ABCC10* promoter upon cisplatin treatment was analyzed with the Mann–Whitney test. Statistically significant differences between analyzed groups were marked with * when *p* < 0.05. Detailed statistical analysis can be found in Table S1. Densitometry result of Western blot images is available in Table S2. Original WB can be found at Figure S1.

Bearing in mind that cisplatin triggers the ATM/ATR signaling pathway, which activates inter alia p53, and that p53 is involved in EP300 recruitment to chromatin under certain conditions, we tested the possible involvement of the tumor suppressor in *ABCC10* overexpression during the development of A549 cell resistance to cisplatin. Neither basal nor drug-resistant cells were characterized by p53 enrichment at the *ABCC10* promoter (Figure 3A). Moreover, transient silencing of the genome guardian did not considerably affect the gene and protein expression (Figure 3B,C). This led to the conclusion that p53 does not control *ABCC10* overexpression under resting conditions in the cisplatin-resistant phenotype.

This prompted us to test the possible contribution of p53 to direct cell response to cisplatin. Cell treatment with an alkylating drug for 24 h caused p53 recruitment to *ABCC10* promoter in non-resistant cells (Figure 3D). To test the functional impact of p53 to EP300 occurrence on the chromatin, we visualized chromatin-bound EP300 by immunostaining in p53-deficient and proficient A549 cells exposed to a single dose of cisplatin for 24 h (Figure 3E). Similar to Figure 2D, confocal images confirmed the higher abundance of acetyltransferase in cisplatin-resistant cells under resting conditions regardless of p53 status. A single dose of cisplatin induced a significant increase in EP300 occurrence on the chromatin only in non-resistant, p53 proficient cells; however, the silencing of p53 entirely precluded acetyltransferase enrichment in the nuclei of basal A549 cells. Notably, cisplatin-resistant cells did not respond to another cisplatin dose with a further increase in enzyme abundance in the nuclei.

To test the functional impact of p53 on the cisplatin-induced expression of *ABCC3* and *ABCC10* in non-resistant A549 cells, we compared their mRNA and protein level in cisplatin-treated cells deficient and proficient in tumor suppressors. We made use of iEP300 to simultaneously monitor the possible cross-talk between p53 and acetyltransferase (Figure 3F–I). As expected, p53 silencing and inhibition of EP300 did not affect the mRNA and protein level of *ABCC3* in cisplatin-treated cells (Figure 3F,G, respectively). While checking the response of *ABCC2* and *ABCC5* to cisplatin in the presence of pan-HDAC inhibitor, we observed p53 dependence (Supplement S2). The silencing of p53 abrogated cisplatin-induced activation of *ABCC2* and *ABCC5* upon deficiency of HDAC activity. Similarly, p53 silencing prevented the cisplatin-induced increase in *ABCC10* transcription, which was only possible in the presence of the active EP300 (Figure 3H,I). These results suggest that p53 is required for EP300-dependent *ABCC10* gene response to the alkylating agent.

### 3.5. CoREST-Free Promoter of ABCC10 Responds to Cisplatin in EP300 and p53-Dependent Fashion in TNBC Cell Line—MDA-MB-231

To confirm that the above-described mechanism controls ABCC gene expression in other cancer types, which are also eligible for cisplatin-based chemotherapy, we tested the triple-negative (progesterone, estrogen, and HER2 deficient; TNBC) breast cancer cell line MDA-MB-231 in a similar experimental model. We generated a corresponding cisplatin-resistant phenotype and measured ABCC gene responsiveness to alkylating drug treatment. Following another paper on this cell line by Strachowska et al., we chose *ABCC4* for further experiments, which was comparably transcribed in cisplatin-resistant and non-resistant phenotypes and in *ABCC10*, which was substantially overexpressed in cells characterized by decreased vulnerability to anticancer drugs.

**Figure 4:**
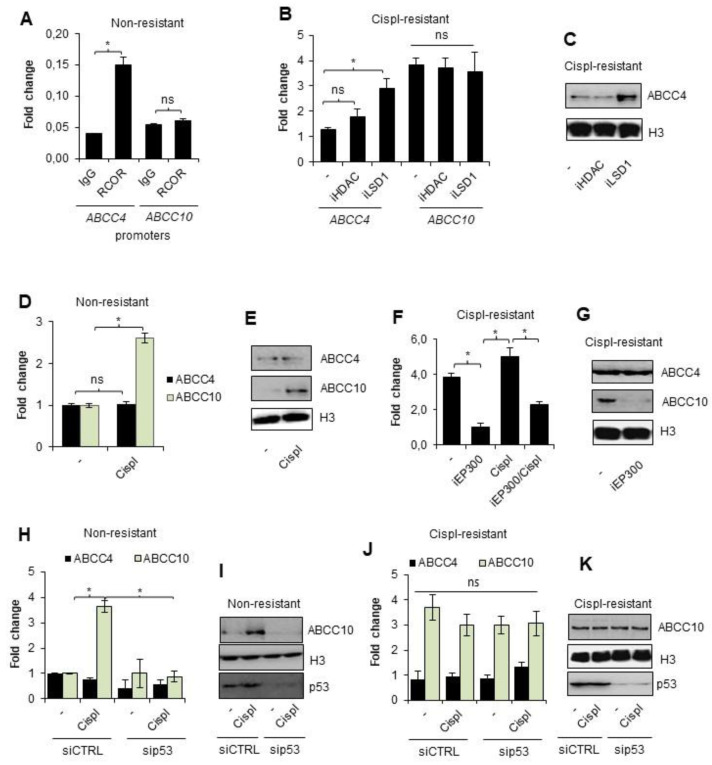
Overexpression of *ABCC10* in cisplatin-resistant MDA-MB-231 cells is controlled by EP300 that activates *ABCC10* transcription in p53-dependent fashion during the direct response of non-resistant breast cancer cells to cisplatin. (**A**) Enrichment of CoREST protein at the promoters of *ABCC4* and *ABCC10* was studied by ChIP-qPCR. Ten percent input was used as an internal control. (**B**) Response of *ABCC4* and *ABCC10* to HDAC and LSD1 inhibition was tested by measurement of mRNA level in cisplatin-resistant cells exposed to the action of iLSD1 (0.1 µM SP2509) and iHDAC (250 µM sodium butyrate) for 24 h. mRNA of ABCC genes was quantified by TaqMan-based real-time PCR and normalized to *ACTB*, *GAPDH* and *TBP*, and the gene expression of non-resistant untreated cells was set as 1. (**C**) A similar approach was employed to assess iLSD1 and iHDAC effect on ABCC4 and ABCC10 protein level, which was visualized by Western blot. Histone H3 was taken as a loading control. (**D**) Activation of *ABCC4* and *ABCC10* by short treatment of non-resistant cells with cisplatin (10 µM) for 24 h was studied by quantifying their mRNA level by TaqMan-based real-time PCR. After normalization to housekeeping genes (*ACTB*, *GAPDH*, *TBP*), gene expression was referred to untreated control that was assumed as 1. (**E**) Response of *ABCC10* to 24 h exposure of non-resistant cell to cisplatin (10 µM) was confirmed by visualizing ABCC10 protein by Western blot. Histone H3 was used as a control. (**F**) TaqMan-based real-time PCR was utilized to confirm the contribution of EP300 to overexpression of *ABCC10* in cisplatin-resistant MDA-MB-231 cell line. Breast cancer cells were added with iEP300 (5 µM C646) for 24 h. Simultaneously, the impact of 10 µM cisplatin on *ABCC10* transcription alone and in combination with iEP300 was tested. In this experiment, iEP300 was added to cells for 1 h prior to cisplatin. Gene transcription was normalized to *ACTB*, *GAPDH*, and *TBP* and the value for untreated cisplatin-resistant cells was assumed as 1. (**G**) The impact of EP300 inhibitor—5 µM C646—added to cisplatin-resistant cells on expression of ABCC10 was confirmed at the protein level by Western blot. ABCC4 served and non-responding genes and histone H3 were used as a loading control. (**H**–**K**) Impact of p53 on induction of *ABCC4* and, particularly, *ABCC10* by 10 µM cisplatin added to non-resistant and cisplatin-resistant MDA-MB-231 cells was tested by applying p53 targeted siRNA. Cells were transfected in parallel with siCTRL and sip53, and after 48 h, cisplatin was administrated to cells for another 24 h. Gene transcription was measured by TaqMan-based real-time PCR (non-resistant cells—H, cisplatin resistant—J) as described in previous experiments and protein was visualized by Western blot (non-resistant—I, cisplatin resistant—K). (**A**,**B**,**D**,**F**,**H**,**J**) All bars represent the mean of replicates ± SEM. (A) The difference in RCOR enrichment at *ABCC4* and *ABCC10* was analyzed with the Mann–Whitney and *t* test, respectively. (**B**,**H**) The impact of HDAC and LSD1 inhibition on *ABCC4* and *ABCC10* expression and cisplatin on non-resistant MDA-MB-231 with different p53 levels was evaluated with Kruskal–Wallis test and Dunn’s multiple comparison test. (**D**) The change in expression of *ABCC4* and *ABCC10* was analyzed with *t* test. (**F**) The expression of ABCC10 in cisplatin-resistant MDA-MB-231 was compared the ANOVA with Tukey’s post hoc test. (**J**) Difference between p53-deficient and non-deficient cisplatin-resistant MDA-MB-231 was evaluated with the ANOVA and Tukey’s multiple comparison test. (**A**,**B**,**D**,**F**,**H**,**J**) Statistically significant differences between analyzed groups were marked with * when *p* < 0.05. Detailed statistical analysis can be found in Table S1. Protein bands in Western blot images were quantified by densitometry with ImageJ, and data are included in Table S2. Original WB can be found at Figure S1.

Similar to our observations carried out for *ABCC3* in wild-type A549 cells, the promoter of *ABCC4*, but not *ABCC10*, was also enriched in the Co-REST protein (Figure 4A). To verify if Co-REST enzymatic subunits functionally affect the expression of the two considered genes, cisplatin-resistant MDA-MB-231 cells were treated with inhibitors of HDACs and LSD1 (Figure 4B,C). Similar to A549 cells, the expression of *ABCC10* was not altered by any of the two inhibitors in the resistant MDA-MD-231 cell line, whereas *ABCC4* mRNA and protein levels increased after LSD1 inhibition (Figure 4B,C, respectively). This supports the hypothesis on the crucial role of CoREST in ABCC gene response to cisplatin in the studied TNBC cells (Figure 4B,C).

Non-resistant breast cancer cells augmented the expression of *ABCC10*, but not *ABCC4*, after their incubation with a single dose of cisplatin (Figure 4D,E). Furthermore, EP300 was responsible for *ABCC10* overexpression in the drug-resistant phenotype since the EP300 inhibitor—C646—substantially reduced mRNA and protein levels of *ABCC10* (Figure 4F,G, respectively). Similarly, transcription of this gene was not considerably increased by a single dose of cisplatin. Expression of *ABCC4* expression remained at the same level regardless of acetyltransferase inhibition.

Having confirmed EP300 as an activator of *ABCC10* transcription under cisplatin-induced stress, in the next step, we tested the mutual interdependence between p53 and EP300-mediated expression of this gene in both phenotypes of MDA-MB-231 cells. Similar to lung cancer, p53 was necessary for EP300-mediated induction of *ABCC10* expression by cisplatin (Figure 4H,I). Transient silencing of p53 did not allow for the increase in EP300-dependent gene transcription in MDA-MB-231 non-resistant cells. Moreover, none of the tested conditions had a significant impact on ABCC4 expression. Furthermore, deficiency of p53 did not alter *ABCC10* transcription in cisplatin-resistant cells, thereby suggesting that this protein is crucial for the direct gene response to cisplatin (Figure 4J,K).

Overall, these data suggest that EP300 acts as a key activator of *ABCC10* in cisplatin-treated MDA-MB-231 cells but requires p53 to trigger direct *ABCC10* transcription in response to the drug.

## 4. Discussion

Our study demonstrates a novel mechanism that involves cooperation between EP300 and p53 and explains differences in expression of ABC family transporters between non-resistant and cisplatin-resistant NSCLC and TNBC cells. We provide experimental evidence that exposure of non-small cell lung and triple-negative breast cancer cells to cisplatin is associated with the recruitment of acetyltransferase and p53-dependent activation of the *ABCC10* promoter. The product of this gene was shown to confer resistance to a variety of drugs, including taxanes, nucleoside analogs, epothilone B, Vinca alkaloids, anthracyclines, but also to gefitinib, which acts as an epidermal growth factor receptor tyrosine kinase inhibitor (EGFR-TKI) that is often considered clinically as first-line therapy in patients with advanced non-small cell lung cancer (NSCLC) with EGFR-activating mutations [34,35]. Although *ABCC10* was shown to be highly expressed in non-small cell lung cancer cells and serves as a predictive marker for multi-drug resistance, our results show that it can be further elevated by cancer cell exposure to an alkylating drug—cisplatin [34,36]. Despite over two decades of comprehensive research on the topic of multidrug resistance, or in particular, gained during cisplatin-based chemotherapy, this cancer cell feature remains elusive and is assigned to various pathways, machineries, and biological molecules. According to the current state of knowledge, abnormal patterns of epigenetic changes, such as methylation and acetylation, can be responsible for the development of multidrug resistance in cancer. An altered methylation pattern was detected in the promoters of *ABCB1*, *ABCC1*, and *ABCG2* in breast and lung cancer, whereas the defective activity of histone acetylases including EP300, CBP, and PCAF was reported to be implicated in *ABCB1* overexpression [37,38]. These findings underline the necessity of a deep understanding of epigenetic landscapes, which drives the formation of multi-drug-resistant phenotypes in human tumors, to successfully target drug-resistant phenotypes. In a relatively recent report, EP300 bromodomain inhibitor, I-CBP112, was shown to decrease the expression of *ABCC1*, *ABCC3*, *ABCC4*, *ABCC5*, and *ABCC10* transporters in MDA-MB-231 cells [27]. Similarly, knockdown of EP300 decreased the expression of *ABCC1* and *ABCG2* in TNBC cells, thereby suggesting the crucial role of the acetyltransferase interaction with chromatin on the high transcription efficacy of some ABC gene family members associated with neoplastic transformation. Considering our current study, EP300 emerges as a key, chromatin-bound enzyme responsible for *ABCC10* overexpression in cisplatin-resistant cancer cells of different tissue origin, e.g., lung and breast [39]. Hence, the above-mentioned enzyme may be considered in the future as a target for breaking multi-drug resistance in some types of cisplatin-resistant cancers. To date, C646 has been documented to transcriptionally repress tumor formation in breast and pancreatic cancer growth by suppressing cyclin B1 and CDK1 [40,41]. Depending on its target and tissue, EP300 plays different roles in tumorigenesis and multi-drug resistance. Zhou et al. proposed a mechanism of EP300 downregulation by miR-106b∼25, which increased tolerance to doxorubicin and helped to avoid doxorubicin-induced senescence in breast cancer cells [42]. In turn, increased activity of EP300 correlated with enhanced resistance to a variety of compounds in prostate cancer [43,44,45]. In breast cancer cells, inhibition of acetyltransferase sensitized cancer cells to a variety of chemotherapeutics, by impeding drug efflux and increasing drug accumulation [27].

Our current study provides evidence on the role of EP300 in cancer cell response to cisplatin and the possible involvement of this enzyme in gaining a multidrug-resistant phenotype of A549 and MDA-MB-231 cell lines. Such a hypothesis is supported by the fact that EP300 inhibitor prevents the cisplatin-induced direct increase in *ABCC10* transcription and reduces higher *ABCC10* expression in the cisplatin-resistant phenotype. In the initial phase of development of cell resistance to cisplatin and other chemotherapeutics, which in our model, was triggered by cancer cell exposure to the alkylating drug, acetyltransferase is recruited to the gene promoter of *ABCC10*, where it facilitates gene expression and maintains it at a higher level in drug-resistant cells, which are generally characterized by EP300-enriched chromatin. The observed higher abundance of the enzyme in nuclei of cisplatin-resistant phenotypes may further suggest that EP300 supports the loss of cancer cell vulnerability to chemotherapeutics by controlling the transcription of numerous genes other than *ABCC10*, which allows cells to overcome anticancer drug toxicity or influx. Such an option may be supported by the study that was focused on the antiapoptotic *BCL2* gene, which emerged transcriptionally to be controlled by EP300 in cardiac myocytes, whereas the CtBP1–p300–FOXO3a complex acts as a transcriptional repressor of the apoptotic regulators Bax and Bim in human osteosarcoma cells [46,47].

EP300 activity was insufficient to increase *ABCC10* transcription in response to the alkylating agent upon p53 deficiency; however, the latter protein was dispensable for the maintenance of high gene transcription in cisplatin-resistant cells. This suggests that p53 might be necessary for the recruitment of EP300 to the gene promoter but does not assist the interaction of the enzyme with chromatin after binding to DNA. Such a possibility seems to be supported by confocal imaging of immunostained acetyltransferase in non-resistant A549 cells exposed to cisplatin, where strong enrichment of chromatin-bound EP300 was only observed in a p53 proficient background; however, p53 deficiency did not affect higher acetyltransferase abundance in the nuclei of the cisplatin-resistant phenotype. In line with our findings, numerous other papers describe p53 as a driver of cell adaptation to the alkylating agent. Cisplatin-resistant squamous head and neck carcinoma that bears cytoplasm-accumulated mutant p53—but not with mutant p53—accumulated in the nucleus and overexpressed glutathione, *ABCC2*, and *ABCG2* [48]. p53 was also reported to increase drug efflux independently from *ABC*-transcription regulation. Together with the Rab-coupling protein, this tumor suppressor was reported to facilitate *ABCB1* reconstitution to the plasma membrane, thereby increasing cisplatin efflux [49]. The role of p53 in the development of cell resistance to cisplatin goes beyond alteration in drug efflux. This protein was reported to contribute to apoptosis evasion of NSCLC cells H1299 by interacting with caspase 9, hence preventing its cleavage and activation [24]. In RAS-mutant ovarian cancer, p53 induces HDAC4 phosphorylation and cytoplasmic translocation, therefore increasing the expression of Atg3, Atg12, and LC3B, in turn promoting cisplatin-induced apoptosis escape via autophagy [50].

In our model, p53 emerged indispensable for EP300-dependent increase in *ABCC10* in non-resistant NSCLC and TNBC cells treated with a single dose of cisplatin. This might be mechanistically explained by the documented involvement of p53 in the acetylase interaction with DNA. Both mutant and wild-type tumor suppressors were shown to activate the acetyltransferase activity of p300 through the enhancement of p300 autoacetylation, which resulted in the accumulation of the enzyme near the transcription start sites and the enrichment of transcription-activating histone marks [26]. Furthermore, cisplatin-induced recruitment of EP300 by p53 has already been reported; Liu et al. observed EP300/CITED2 enrichment at the promoter of *ERCC1* that was mediated by p53 after cell exposure to an alkylating drug [51]. Of note, ERCC1 acts as an endonuclease responsible for the 5′-incision during DNA repair. These findings suggest that the protein, which is known as a tumor suppressor, may help cancer cells gain a drug-resistant phenotype. Interdependence between EP300 and p53 occurs in both tested cell lines, regardless of their p53 status. A549 cells express wild-type p53, whereas MDA-MB-231 cells possess single R280K mutants [52]. This natively occurring gain of function mutation in MDA-MB-231 cells correlates with the number of cancer cell processes, including proliferation, metastasis and apoptosis evasion [53,54]. The missense mutation limits interactions between the protein and DNA by removing two hydrogen bonds that are formed between R280 and the DNA in wild-type p53, potentially decreasing its activity [55,56]. According to the study by Vrba et al., due to limited DNA binding ability, R280K p53 mutant selectively recognizes transcriptionally active promoters with high levels of acetylation [57]. Assuming that in our ABCC genes, which are actively transcribed upon cisplatin treatment or in cisplatin-resistant phenotype, one may expect that promoters of target ABCC genes are transcriptionally permissive and possess high levels of acetylation. Therefore, even the R280K mutant may interact with these regions. Increased affinity of p53 toward transcriptionally permissive genomic regions, which is caused by the gain of function mutation, may additionally stimulate ABCC gene expression and, hence, enhance chemoresistant phenotype of cancer cells. Conversely, considering the relevance of promoter acetylation that is vital for the R280K p53 mutant to bind the DNA, inhibition of Ep300 may be considered as an alternative treatment against cancers possessing the p53 mutation, by hampering promoter acetylation and thus mutant p53 binding to the target promoters.

Another aspect that deserves attention is the chromatin architecture at the gene promoters that allows for or interferes with p53-dependent gene activation by EP300 in response to cisplatin. In both considered cell lines, non-small cell lung cancer and triple-negative breast cancer, the promoters of genes, which were not overexpressed in the cisplatin-resistant phenotype, were enriched in components of the CoREST repressive complex such as CoREST (RCOR1—REST corepressor 1) and enzymatically active subunits: HDAC1 and LSD1. The last two enzymes were successfully targeted with their inhibitors, which unlocked suppressed transcription of *ABCC3* and *ABCC4* in A549 and MDA-MB-231 cell lines, respectively. Although CoREST protein was detected at the above-mentioned gene promoters, the dominant role of HDAC1 and LSD1 in gene repression varied between the two cell lines. LSD1 inhibitor increased transcription of *ABCC4*, whereas the effect of pan-HDAC inhibitor was observed for *ABCC3*. This may be explained by the presence of various transcription coactivators or histone marks (particularly K4meK9ac, K4meK9, and K4K9ac) at the gene promoters since the cross-talk between LSD1 and HDAC1 is shaped by the substrate availability [31]. The role of HDAC and LSD1-dependent gene repression in the regulation of drug resistance in cancer has already been documented. Wang et al. observed increased expression of the *ABCC10* transporter, while the expression of *ABCC3* was lowered in A549 and HCT116 cells after iHDACs (SAHA and TSA) treatment [58]. In a study by Shi et al., sodium butyrate sensitized HCT116 and A549 cells to chlorambucil; however, simultaneously, resistance to 5-fluorouracil was enhanced [59]. This suggests that the effect of HDAC inhibitors may depend on the drug that the inhibitor is combined with or on the specificity of the HDAC inhibitor since the referred sodium butyrate lacks specificity toward any particular HDAC. Moreover, the effect of simultaneous deficiency of two or more HDACs activity may vary from inhibition of a single enzyme. The other CoREST complex member, LSD1, was reported to repress *ABCC1* and *ABCC10* gene expression upon inhibition of EP300 in the triple-negative breast cancer cell line MDA-MB-231 [27]. Bearing in mind that the promoter of *ABCC10* is not repressed by LSD1 and HDACs in MDA-MB-231 and A549 cells, nor enriched in CoREST protein, the deficiency of EP300 activity seems to recruit histone demethylase or the entire CoREST complex to the *ABCC10* gene regulatory element. Moreover, inversely, the lack of the CoREST repressive complex at the *ABCC10* promoter allows for EP300 binding and transcription enhancement in cancer cells treated with cisplatin. Therefore, the CoREST complex seems to mark promoters of *ABCC* genes, which remain irresponsive to stimulation with cisplatin. Considering our findings, CoREST distribution in the genome may be considered as a predictive marker of *ABCC* gene response to cisplatin-based chemotherapy. Specific inhibitors of inducible membrane transporters may be combined with alkylating drugs in future work to overcome multi-drug resistance caused by increasing the expression of the CoREST-free ABCC transporter during chemotherapy. Furthermore, other areas are worth considering with possible clinical perspectives. These include CoREST involvement in the development of cancer cell resistance to other chemotherapeutics, particularly anthracyclines, the contribution of this repressive complex to modulate other cancer-promoting gene expressions during chemotherapy, and the applicability of the above-described CoREST-p53-EP300 interdependence to p53-mutated tumors.

## 5. Conclusions

In summary, our study reveals a novel interdependence between CoREST occurrence at the *ABCC* gene promoters and their responsiveness to cisplatin. We provide an insight into p53-dependent transcriptional activation of CoREST-free *ABCC10* by EP300 in cells exposed to an alkylating drug. Acetyltransferase is responsible for higher *ABCC10* expression in cisplatin-resistant non-small cell lung and triple-negative breast cancer cell lines. The described regulatory mechanism may be of particular importance for future anti-cancer treatment approaches involving cisplatin.

## Data Availability

The data presented in this study are available in the supplementary material Table S1.

## Supplementary Materials

The following supporting information can be downloaded at: https://www.mdpi.com/article/10.3390/cancers14040894/s1, Figure S1: Full Western blot images; Figure S2: p53-dependent response of *ABCC2* and *ABCC5* to cisplatin; Table S1: Raw data and statistical analysis. Table S2: Densitometry readings/ratios for all presented Western blot images.
