# Supplementary material for: Activation of ABCC Genes by Cisplatin Depends on the CoREST Occurrence at Their Promoters in A549 and MDA-MB-231 Cell Lines
Source: Cancers (Basel). 2022 Feb 11;14(4):894. doi: 10.3390/cancers14040894 (PMC8870433; doi:10.3390/cancers14040894)
Supplement: Supplementary file 1 [file cancers-14-00894-s001.zip › Figure S1.pptx]

## Slide 1
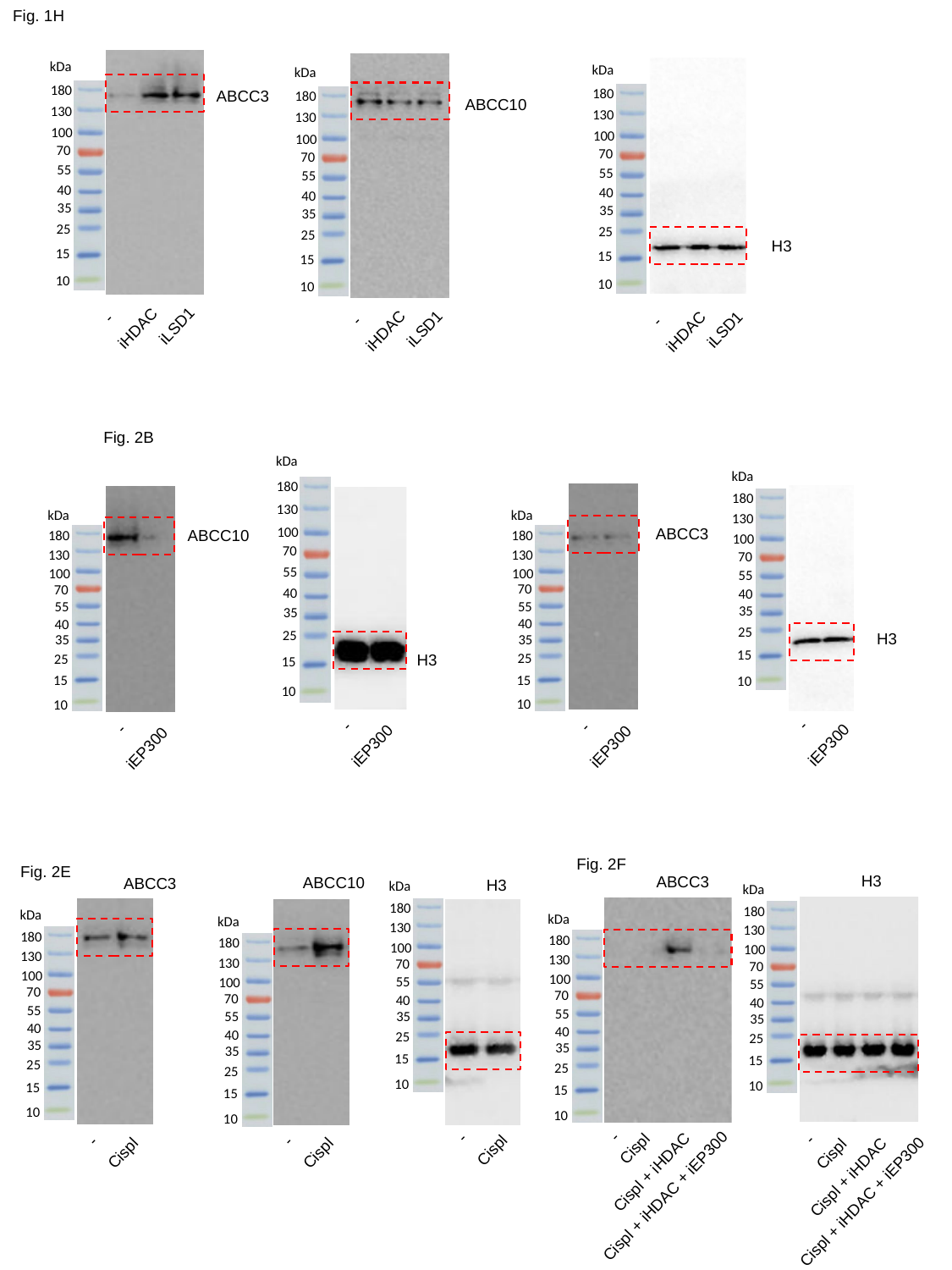

Fig. 1H
-
iLSD1
iHDAC
ABCC3
kDa
180
130
100
70
55
40
35
25
15
10
kDa
180
130
100
70
55
40
35
25
15
10
kDa
180
130
100
70
55
40
35
25
15
10
ABCC10
H3
-
iLSD1
iHDAC
-
iLSD1
iHDAC
Fig. 2B
kDa
180
130
100
70
55
40
35
25
15
10
kDa
180
130
100
70
55
40
35
25
15
10
H3
-
iEP300
ABCC10
-
iEP300
-
iEP300
H3
kDa
180
130
100
70
55
40
35
25
15
10
kDa
180
130
100
70
55
40
35
25
15
10
ABCC3
-
iEP300
Fig. 2F
Fig. 2E
H3
ABCC3
ABCC10
-
Cispl
ABCC3
H3
-
Cispl
kDa
180
130
100
70
55
40
35
25
15
10
kDa
180
130
100
70
55
40
35
25
15
10
kDa
180
130
100
70
55
40
35
25
15
10
kDa
180
130
100
70
55
40
35
25
15
10
kDa
180
130
100
70
55
40
35
25
15
10
Cispl
Cispl + iHDAC
Cispl + iHDAC + iEP300
Cispl
Cispl + iHDAC
Cispl + iHDAC + iEP300
-
Cispl
-
-

## Slide 2
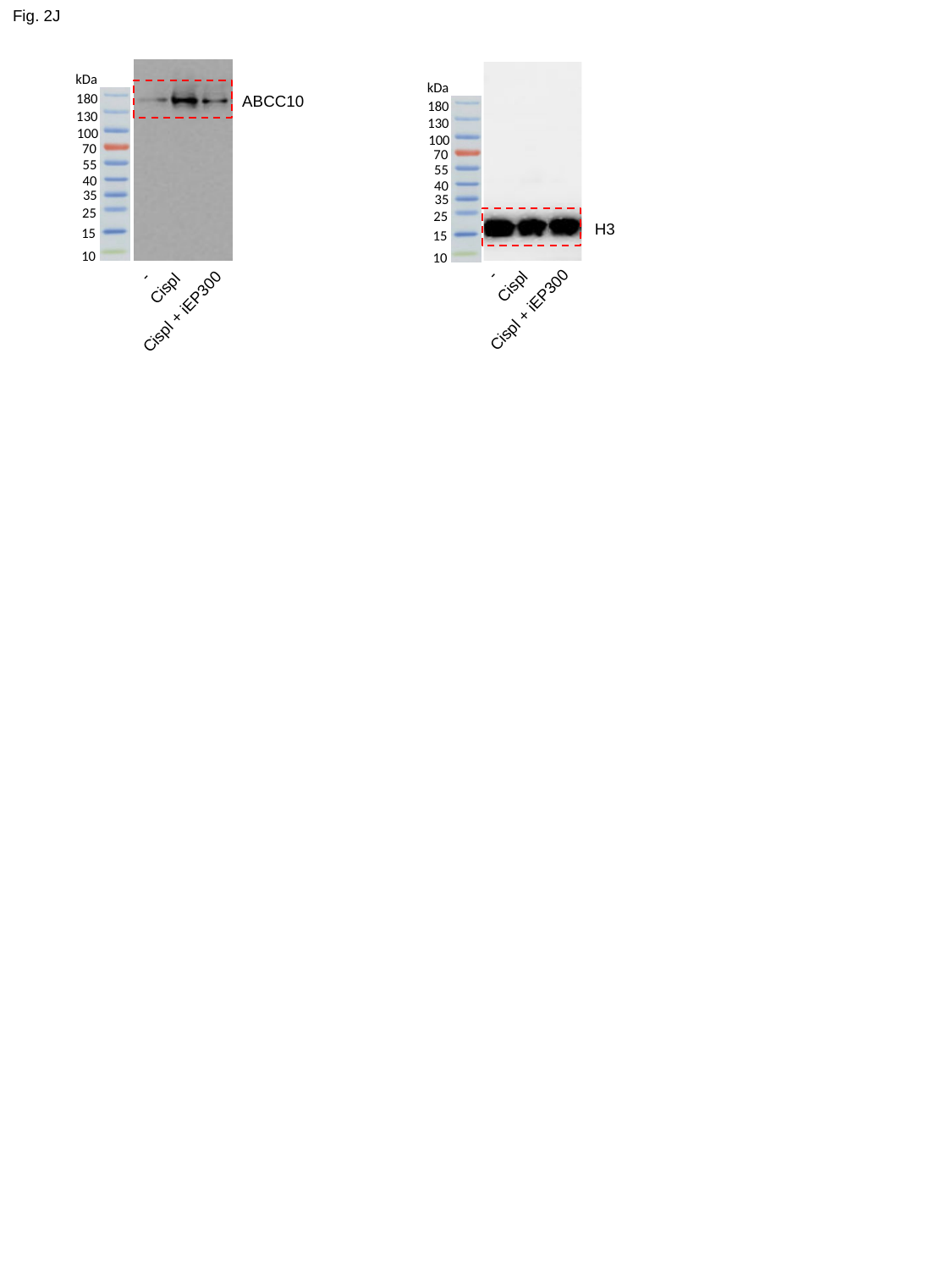

Fig. 2J
ABCC10
-
Cispl
Cispl + iEP300
H3
-
Cispl
Cispl + iEP300
kDa
180
130
100
70
55
40
35
25
15
10
kDa
180
130
100
70
55
40
35
25
15
10

## Slide 3
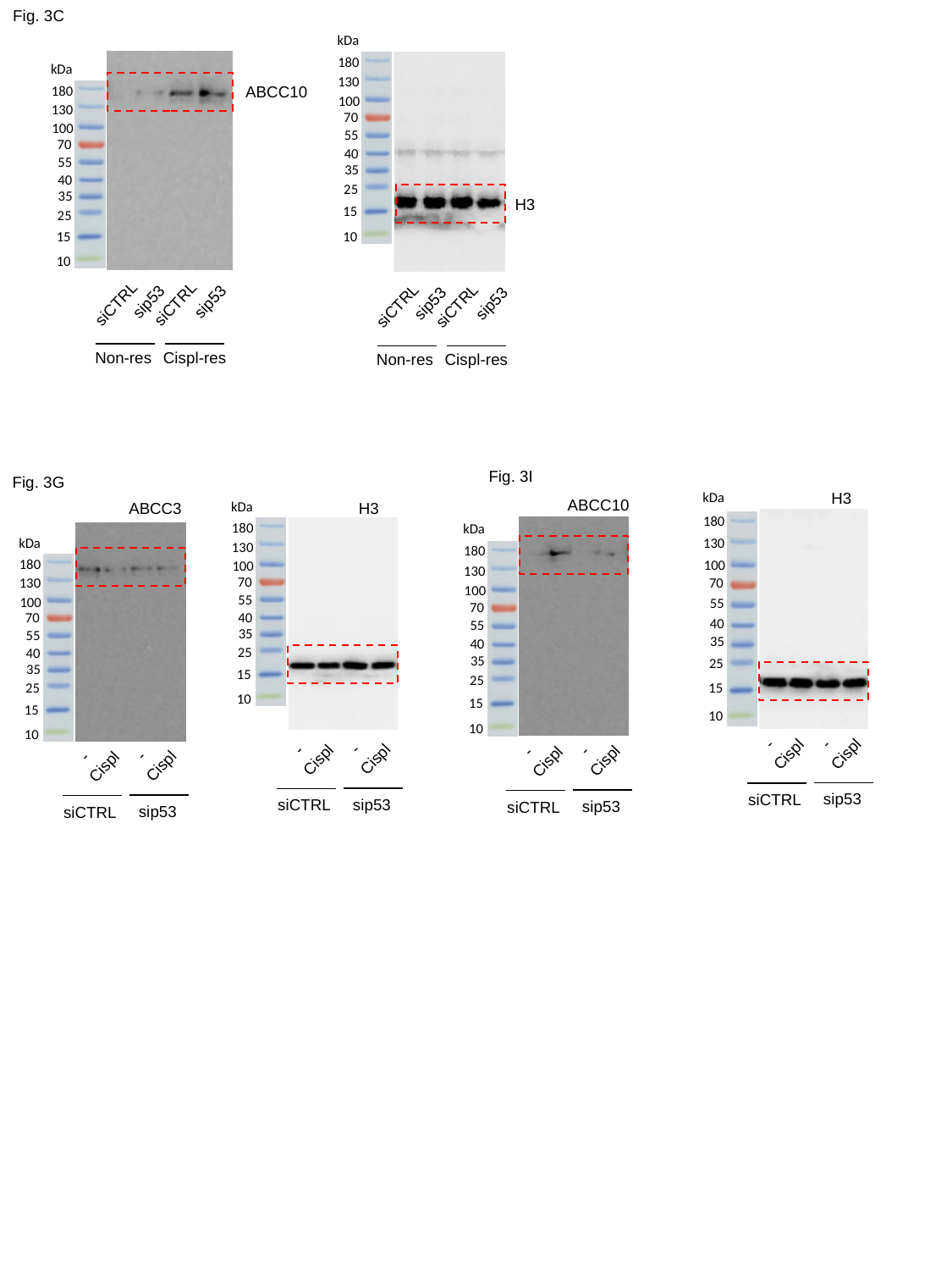

Fig. 3C
kDa
180
130
100
70
55
40
35
25
15
10
H3
sip53
sip53
siCTRL
siCTRL
Non-res
Cispl-res
ABCC10
sip53
sip53
siCTRL
siCTRL
Non-res
Cispl-res
kDa
180
130
100
70
55
40
35
25
15
10
Fig. 3I
Fig. 3G
kDa
180
130
100
70
55
40
35
25
15
10
H3
ABCC10
-
Cispl
-
Cispl
sip53
siCTRL
kDa
180
130
100
70
55
40
35
25
15
10
ABCC3
-
Cispl
-
Cispl
sip53
siCTRL
H3
-
Cispl
-
Cispl
sip53
siCTRL
kDa
180
130
100
70
55
40
35
25
15
10
kDa
180
130
100
70
55
40
35
25
15
10
-
Cispl
-
Cispl
sip53
siCTRL

## Slide 4
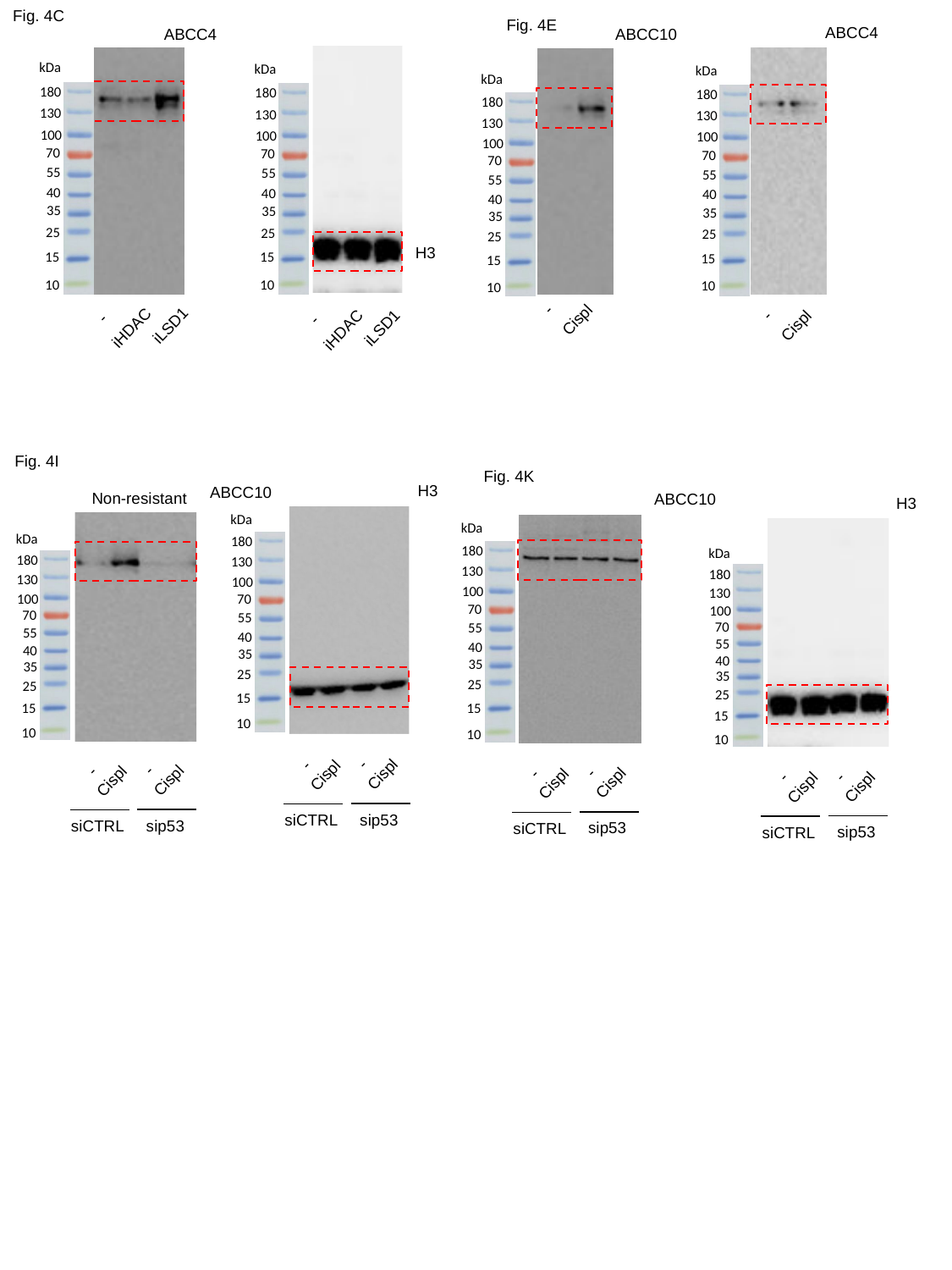

Fig. 4C
Fig. 4E
ABCC4
-
Cispl
ABCC4
-
iLSD1
iHDAC
ABCC10
-
Cispl
H3
-
iLSD1
iHDAC
kDa
180
130
100
70
55
40
35
25
15
10
kDa
180
130
100
70
55
40
35
25
15
10
kDa
180
130
100
70
55
40
35
25
15
10
kDa
180
130
100
70
55
40
35
25
15
10
Fig. 4I
Fig. 4K
H3
-
Cispl
-
Cispl
sip53
siCTRL
ABCC10
Non-resistant
-
Cispl
-
Cispl
sip53
siCTRL
ABCC10
-
Cispl
-
Cispl
sip53
siCTRL
H3
-
Cispl
-
Cispl
sip53
siCTRL
kDa
180
130
100
70
55
40
35
25
15
10
kDa
180
130
100
70
55
40
35
25
15
10
kDa
180
130
100
70
55
40
35
25
15
10
kDa
180
130
100
70
55
40
35
25
15
10
