# Supplementary material for: Activation of ABCC Genes by Cisplatin Depends on the CoREST Occurrence at Their Promoters in A549 and MDA-MB-231 Cell Lines
Source: Cancers (Basel). 2022 Feb 11;14(4):894. doi: 10.3390/cancers14040894 (PMC8870433; doi:10.3390/cancers14040894)
Supplement: Supplementary file 1 [file cancers-14-00894-s001.zip › Figure S2.pptx]

## Slide 1
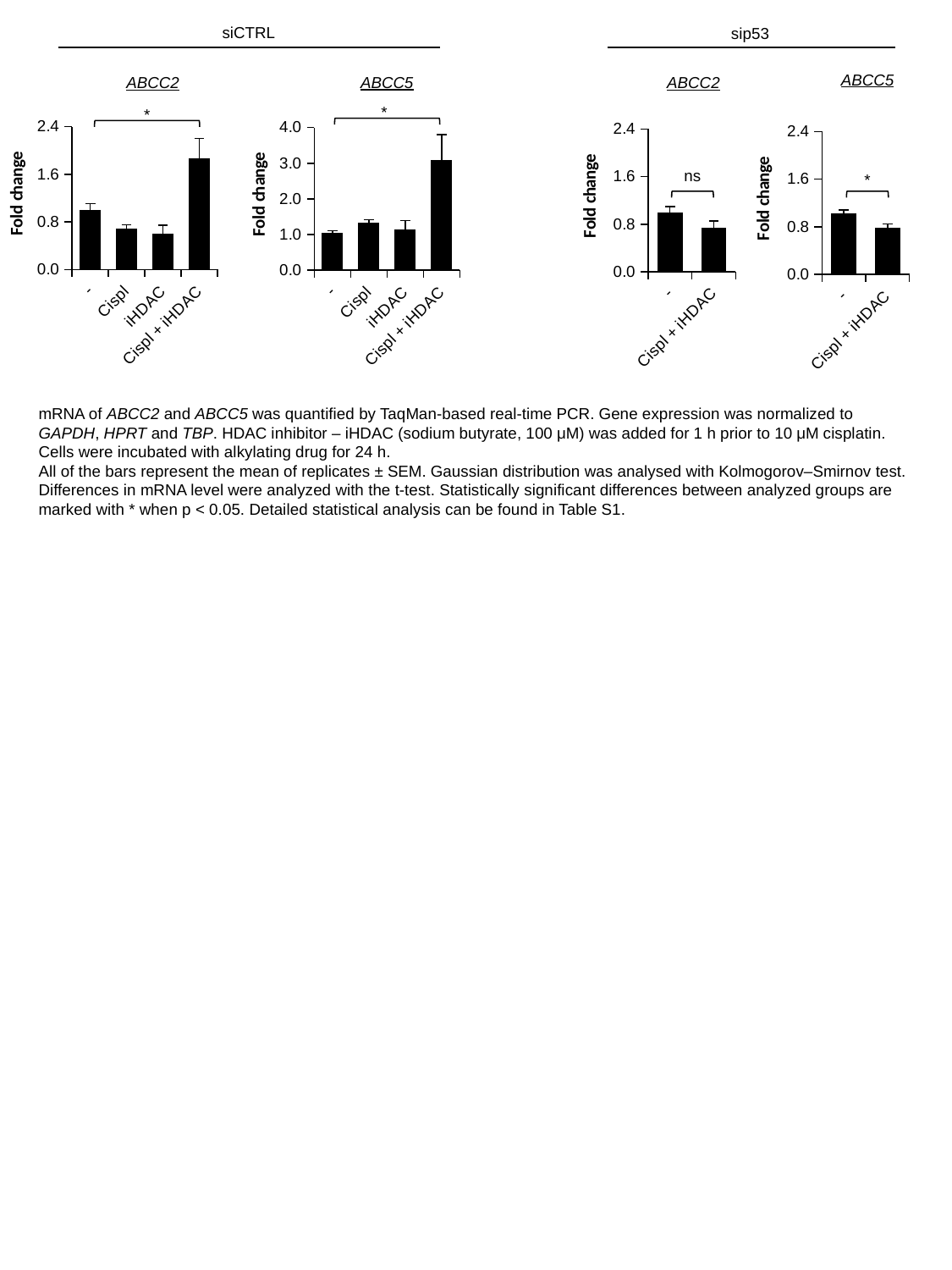

siCTRL
sip53
ABCC5
ABCC5
ABCC2
ABCC2
### Chart
| Category | |
|---|---|
| - | 0.9993317603419959 |
| Cispl | 0.6851288787771533 |
| iHDAC | 0.598697521623295 |
| Cispl + iHDAC | 1.8635781649979695 |
### Chart
| Category | |
|---|---|
| - | 1.031518498479049 |
| Cispl | 1.3171117861087185 |
| iHDAC | 1.1258580082902252 |
| Cispl + iHDAC | 3.0878250864038073 |
### Chart
| Category | |
|---|---|
| - | 0.9924084068848753 |
| Cispl + iHDAC | 0.7350725062509139 |*
### Chart
| Category | |
|---|---|
| - | 1.0147160322339739 |
| Cispl + iHDAC | 0.7752550636204258 |*
ns
*
mRNA of ABCC2 and ABCC5 was quantified by TaqMan-based real-time PCR. Gene expression was normalized to GAPDH, HPRT and TBP. HDAC inhibitor – iHDAC (sodium butyrate, 100 μM) was added for 1 h prior to 10 μM cisplatin. Cells were incubated with alkylating drug for 24 h.
All of the bars represent the mean of replicates ± SEM. Gaussian distribution was analysed with Kolmogorov–Smirnov test.
Differences in mRNA level were analyzed with the t-test. Statistically significant differences between analyzed groups are marked with * when p < 0.05. Detailed statistical analysis can be found in Table S1.
